# Virologic outcomes of people living with human immunodeficiency virus who started antiretroviral treatment on the same-day of diagnosis in Ethiopia: A multicenter observational study

**DOI:** 10.1371/journal.pone.0257059

**Published:** 2021-09-03

**Authors:** Ismael Ahmed, Meaza Demissie, Alemayehu Worku, Salem Gugsa, Yemane Berhane

**Affiliations:** 1 University of Gondar, Gondar, Ethiopia; 2 Addis Continental Institute of Public Health, Addis Ababa, Ethiopia; 3 Department of Preventive Medicine, School of Public Health, College of Health Sciences, Addis Ababa University, Addis Ababa, Ethiopia; 4 Department of Global Health, University of Washington Seattle, Seattle, WA, United States of America; Azienda Ospedaliera Universitaria di Perugia, ITALY

## Abstract

**Introduction:**

There have been tremendous achievements in scaling-up antiretroviral therapy (ART) for treatment of human immunodeficiency virus (HIV), following universal “test and treat” policy implementation in low- and middle-income countries. However, its effects on virologic outcomes is not yet well investigated. We compared low viral load status in people living with HIV between those who were initiated on ART on the same-day and after 7 days of being diagnosed with HIV infection.

**Methods:**

We conducted a retrospective cohort study of persons age ≥15 years-old who were newly diagnosed and started on ART between October 2016 and July 2018 at 11 public health facilities in northwest Ethiopia. Exposure was initiation of ART on the same-day of HIV diagnosis. The outcome was low viral load at 12-months following ART initiation. We used double-robust estimator using inverse-probability-weighted regression adjustment to compare the groups.

**Results:**

A total of 398 people who started ART on the same-day of HIV diagnosis and 479 people who started 7 days after the initial diagnosis were included in this study. By 12-months following ART initiation, 73.4% (292) in the same-day group vs 83.7% (401) in the >7 days group achieved low viral load (absolute difference = 10.3% (95% CI: 4.9%, 15.8%)). After adjusting for baseline and follow-up covariates, there was statistically significant difference in low viral load status (adjusted difference = 8.3% (95% CI: 3.5%, 13.0%)) between the same-day group and the >7 days group.

**Conclusions:**

Achievement of low viral load by 12-months post-initiation of ART was not optimal among participants who started ART on the same-day of HIV diagnosis. Efforts should be made to reinforce treatment adherence while initiating same-day ART.

## Introduction

The large scale-up of antiretroviral therapy (ART) to treat human immunodeficiency virus (HIV) infections in low- and middle-income countries has resulted in significant gains in improving the life of people living with HIV. It has significantly reduced new infections and Acquired Immunodeficiency Syndrome (AIDS)-related deaths [1]. As part of improving global ART coverage, the Joint United Nations Programme on HIV and AIDS (UNAIDS) launched global goals towards ending the HIV epidemic through supporting country- and region-led efforts to establish new targets for HIV treatment scale-up in October 2014. This ambitious 90-90-90 target aimed to achieve: 90% of all people living with HIV to know their HIV status, 90% of all people with diagnosed HIV infection to receive sustained ART, and 90% of all people receiving ART to achieve viral suppression by 2020 [2]. UNAIDS released a revised “fast-track targets” to achieve 95-95-95 goals for countries to end the AIDS epidemic by 2030 [3].

Attaining viral suppression is the primary goal of ART initiation thereby preventing HIV-related morbidity and mortality through restoration of immunologic function [4, 5]. The 2021 World Health Organization (WHO) guideline recommends a threshold of 50 copies/mL for viral load suppression after ART initiation as a public health approach [6]. ART initiation is also considered as HIV prevention strategy due to reduced infectiousness of the person as a result of reduced amount of virus in the blood to undetectable level. A systematic review on the risk of HIV transmission among serodiscordant partners reported that there is a negligible risk of sexual transmission of HIV when an HIV-positive sex partner adheres to ART and maintains a suppressed viral load of <200 copies/mL on consecutive measurements every 4 to 6-months [7]. These scientific evidences established Undetectable = Untransmittable (U = U) concept of HIV prevention which applies treatment for HIV as a powerful strategy to prevent HIV transmission [8]. Furthermore, a recent observational study that followed individuals who had reached undetectable viral load status (≤200 copies/mL for >6 months) showed that most of them had maintained undetectable viral load level which is essential for sustained HIV prevention [9].

In response to new scientific developments that showed favorable clinical outcomes of early ART initiation including better viral load suppression [10, 11], the WHO released a recommendation to provide universal “test and treat” services for people living with HIV in 2016, regardless of their CD4 cell count or WHO clinical stage [12]. Ethiopia endorsed this policy and started its implementation in August 2016 [13]. Though there have been efforts made to link persons diagnosed with HIV to immediate ART initiation services including same-day treatment, access to routine viral load monitoring at 6-months, at 12-months and every 12-months thereafter, following ART initiation has been a challenge in clinical care settings. Program data shows that, in Ethiopia only 76% of people on ART received viral load monitoring services as recommended by the national guideline by end of December 2019 [14].

As rapid ART initiation immediately after HIV diagnosis is a relatively new approach in low-income settings (LIS), there is a scarcity of evidence to evaluate its effectiveness in viral load suppression in routine clinical care settings. Even though two earlier randomized control trials (RCTs) demonstrated the efficacy of same-day ART initiation in improving retention and viral suppression at 12-months [11, 15], recent empirical observational studies raise concerns about whether the benefits measured in RCTs fully translate into real-world settings [16–18]. A recent South African observational study that compared the virologic outcomes of same-day initiators with later initiators identified no difference in viral suppression (<400 copies/mL) at 6-months post-ART initiation [19]. A similar program evaluation in Nigeria [20] that attempted to compare viral suppression rates (≤400 copies/mL) before and after universal “test and treat” policy found no difference in viral suppression rates using the limited viral load data. On the other hand, the observational studies that reported unfavorable retention outcomes among same-day ART initiators were not able to assess the effect of same-day ART initiation on virologic outcomes due to absence of documented viral load result for a significant proportion of participants [16, 17]. This may be due to limited implementation of routine viral load testing services in LIS [21–23].

In general, despite evidence of increased trend of individuals enrolling for rapid ART including same-day initiation in LIS [16, 17, 24], there are limited empirical evidence on virologic outcomes following universal “test and treat” policy implementation. Therefore, in this observational study, we aimed to compare the proportion of low viral load status between people who were initiated on ART on the same-day of HIV diagnosis and those who started treatment >7 days after HIV diagnosis.

## Methods

### Study design

We conducted a retrospective cohort study using routinely collected data from multiple ART clinics to compare virologic outcomes among people who were initiated on same-day ART with those initiated >7 days after HIV diagnosis.

### Study setting

This study was part of the study that evaluates the effectiveness of same-day ART initiation in Ethiopia. The detail of the study setting has been published elsewhere [18]. Briefly, this study was conducted at 11 public health facilities in Bahir Dar and Gondar, towns in Amhara region of northwest Ethiopia. The list of study sites includes: Abay health center, Addis Alem primary hospital, Azezo health center, Bahir Dar health center, Felege Hiwot hospital, Gondar health center, Gondar University hospital, Han health center, Maraki health center, Shimbit health center, and Teda health center. The health facilities provide comprehensive HIV services including diagnosis, care and treatment of HIV and opportunistic infections (OIs) using a multidisciplinary team approach. The health facilities have access to a centralized viral load testing laboratory in each town connected through postal service. People newly diagnosed with HIV infection are immediately linked to ART clinic for treatment initiation following a confirmatory HIV test if they have no sign of OIs that warrant delay in ART initiation and ready to start ART at the initial clinical visit. Those who deferred rapid ART initiation were counseled and given an appointment to return a week following their HIV diagnosis for initiation [25].

### Participants

Adult (≥15 years-old) people living with HIV who reported new HIV infection diagnosis and started ART between October 2016 and July 2018 at participating health facilities were categorized into two groups: those who started ART on the same-day of diagnosis and those started ART after 7 days of the initial diagnosis. We excluded medical records of participants who were initiated on ART 1–7 days after diagnosis, had tuberculosis (TB) or cryptococcal meningitis, aged <15 years old, pregnant, transferred-in from another health facility, and transferred-out to another health facility within 12-months of ART initiation.

### Variables and measurement

The outcome of this study, low viral load status was defined as a documented first viral load result of ≤1000 copies/mL [12] by 12-months of starting ART. We could not measure the viral suppression per the latest WHO definition (<50 copies/mL) [6], since viral load results were not reported using the new threshold in public health facilities during the study period in Ethiopia. The 12-months window period was selected because either the 6- or 12-months viral load tests were missing for some of the participants similar to numerous studies in most LIS [11, 16, 20]. Furthermore, to account for the possibility that routine viral load monitoring is not usually done exactly at 6- or 12-months as per the national guidelines, we included individuals with documented first viral load result any time between 4- to 9-months for the 6^th^ month’s viral load and 10- to15-months for the 12^th^ month’s viral load monitoring following ART initiation [20]. Individuals with undocumented viral load result were excluded from the analysis. However, loss to follow-up (LTFU) individuals with no documented viral load result were considered as having high viral load (>1000 copies/mL) [26–28].

As part of the routine viral load monitoring, blood samples for viral load determination are sent using sample referral form to the respective centralized laboratories in Bahir Dar and Gondar towns. Viral load was measured from plasma specimen by using Abbott Real-Time m2000 and Roche Cobas AmpliPrep/Cobas TaqMan HIV-1 assay analyzer in Amhara Public Health Institute, Bahir Dar town and Gondar University Hospital, Gondar town, respectively.

Covariates included in the analysis were biological (CD4 cell count and body mass index (BMI)), sociodemographic (age, sex, marital status, education, religion, residence, partner’s HIV status and disclosure of HIV status and functional status) and clinical factors (OI, WHO clinical stage, type of antiretroviral (ARV) regimen initiated and taking cotrimoxazole preventive therapy (CPT) and isoniazid preventive therapy (IPT)). We also considered the type of health facility (hospital or health center) where ART services were provided as a covariate.

In addition, adherence to ART was included as an independent factor which was measured by the 6- and 12-months post-initiation of ART. According to the national guidelines, adherence was assessed by a clinician asking a client about the number of missed ARV drug doses during the past month and/or the clinicians’ judgment based on missed clinic follow-up visits. The clinician labels ART adherence as ‘good’ if the person missed ≤2 doses (≥95% adherence), ‘fair’ if the person missed 3–5 doses (85–94% adherence), and ‘poor’ if the person missed ≥6 doses (<85% adherence) out of the 30 doses to be taken during each month of ART follow-up [25]. In this study, individuals with a documentation of ‘good’ adherence at every visit in the last 6-months were categorized as having optimal adherence. Individuals with at least one clinician-recorded ‘poor’ or ‘fair’ adherence measures in any of the last 6-months [29] or individuals who missed ART refill follow-up for a period of ≥30 days between any of the last 6-months [30] were categorized as having sub-optimal adherence at 6- and 12-months.

The description of data sources, methods of data collection and variable measurement in this study has been previously published [18].

### Study size and sampling method

In this study all eligible individuals who were newly diagnosed and started on ART between 20, October 2016 and 18, July 2018 were included, 398 same-day and 497 for the >7 days groups. The estimates were based on results from a South African study [11] with a P_1_ of 64% (proportion of viral suppression at 10-months of ART among persons in the same-day group) and P_2_ of 51% (proportion of viral suppression at 10-months of ART among individuals in the control group). We used a 0.05 significance level, 80% power and a 1:1 proportion. A design effect of 1.5 was used to account for the effect of clustering in this multicenter design. A 15% excess was allowed to compensate for missing medical records which is common in routine services [31]. Using these estimations, we aimed to include 426 participants per group. StatCalc for cohort studies using Epi Info^TM^ version 7 (developed by Centers for Disease Control and Prevention) was used to determine the sample size.

### Statistical analysis

Descriptive statistics were used to summarize sociodemographic and clinical characteristics of study participants including missing data for each variable by group. We compared baseline and follow-up covariates balance between the two groups using χ2 test of equality of proportions.

Missing covariate data were assumed to be missing completely at random (MCAR) based on our knowledge of the data and the HIV program combined with Little’s chi-square MCAR test [32]. We reported the number of missing values for each variable by group. Missing baseline sociodemographic information on patients’ medical record, which is a common problem in HIV care [31, 33], were mainly related to incomplete documentation (accidental skipping) by health care workers. Missing baseline CD4 cell count measurement was due to interrupted CD4 testing service (related to machine failure and stockout of reagent) in most of the health facilities.

We used multiple imputation to improve efficiency in outcome [34] using “multivariate imputation by chained equations (MICE)” method with fully conditional specification (FCS) [35, 36]. We performed augmented-regression for all categorical imputation variables to avoid perfect prediction [35]. Twenty imputed datasets were generated to reduce the sampling error due to imputations based on Stata recommendation [35]. The imputation model included marital status, educational status, religion, place of residence, disclosure of HIV status, partner’s HIV status, baseline CD4 cell count, baseline functional status, adherence to treatment and time to first viral load test. We used Kernel density estimate plots and tabulation of proportions for continuous and categorical variables, respectively to compare the distributions of the observed, imputed, and completed values.

Proportions of individuals achieving low viral load (≤1000 copies/mL) [12] by the first 12-months of starting ART were compared with absolute difference (95% confidence interval (CI)) between the two groups using a χ2 test. We estimated the adjusted effect (average treatment effect among treated participants) using a logistic regression model. We used a double-robust estimator using inverse-probability-weighted regression adjustment (IPWRA) to compensate for any remaining imbalance of covariates in study groups and obtain more precise treatment effect estimates [37, 38]. The inverse-probability weights were generated from propensity scores estimated within each of imputed dataset [39].

We used a backward stepwise variable selection method that included baseline (age, sex, marital status, education, residence, BMI, WHO clinical stage, functional status and OI at enrollment) and follow-up (CPT, IPT, type of ARV regimen initiated, disclosure of HIV status, partner’s HIV status, adherence to treatment and time to first viral load test) covariates to ensure sufficient covariate adjustment. The type of health facility (hospital vs health center) was included to the model as a covariate to adjusted for the effect of health facilities [40]. We explored all possible interaction terms between the main effect variables included in the model and found no significant interaction. The final treatment effect models included: age, WHO clinical stage, functional status, and facility type in the treatment model; and age, WHO clinical stage, IPT and adherence in the outcome model. To assess covariate balance between the study groups, we calculated standardized differences for each baseline covariate included in the final regression model. A standardized mean difference <10% were taken as a cut-off value for covariate balance between the two groups [41]. We reported the adjusted differences (proportions) using a regression coefficient (β) with 95% CI. All statistical analyses were performed using Stata version 13.0 (StataCorp., College Station, TX).

### Sensitivity analysis

In order to understand the viral suppression at early period of ART initiation, we have considered a sensitivity analysis to determine the proportion of individuals with low viral load status by 6-months (viral load test conducted between 4- and 9-months) following ART initiation. We also performed a sensitivity analysis by excluding LTFU individuals (considered as having high viral load in the primary analysis) to observe the effect of treatment only among those who had documented viral load result by 12-months. This sensitivity analysis excluded 131 study participants (79 same-day and 52 >7 days starters) who were LTFU. Finally, in order to compare results based on multiple imputation, we estimated low viral load status by 12-months from analyses restricted to complete-cases (without imputation of covariates) [42].

### Ethical consideration

Ethical clearance was obtained from the Institutional Review Board of the University of Gondar. The ethical clearance with the protocol was shared to Amhara Public Health Institute for further clearance. The institute provided a support letter to town health offices requesting access to the medical records at study sites. Participants were not directly contacted nor were their personal identifiers collected. While reviewing patients’ medical records, non-personal identifiers such as unique ART number or medical record number were used to distinguish study subjects. Only data collectors and supervisors had access to the medical records and both groups signed confidentiality agreements before commencing data collection.

## Results

### Characteristics of study participants

**Fig 1:**
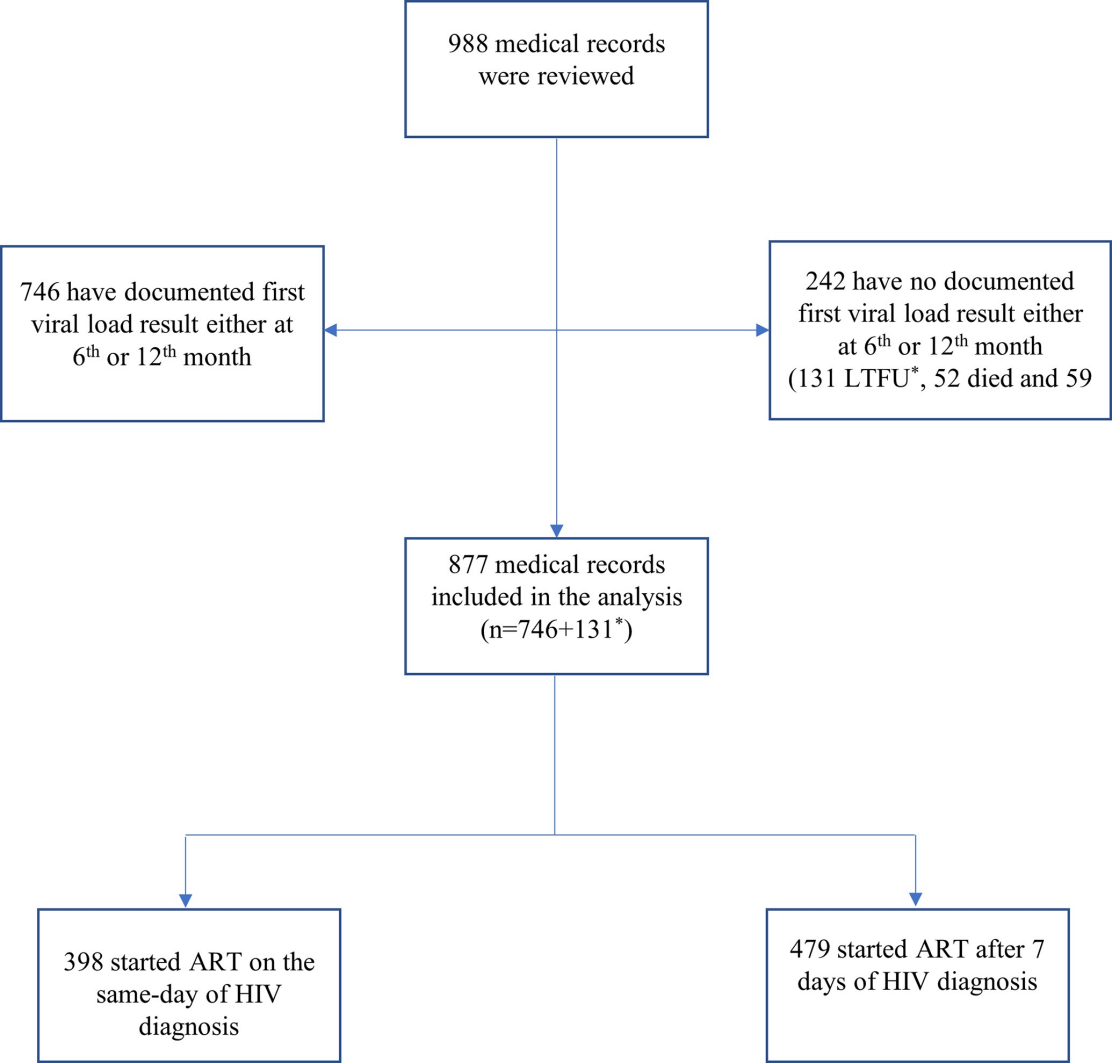
Summary of participants included in the analysis. *LTFU individuals with no documented viral load result were included in the analysis as having unsuppressed viral load.

Of the 988 medical records reviewed, 746 (75.5%) participants (n = 319, 73.7% in the same-day group and n = 427, 76.9% in the >7 days group) had documented first viral load result either for the 6^th^ month’s or 12^th^ month’s follow-up period. Out of the remaining 242 participants who had no documented viral load result, 131 (79 in the same-day group and 52 in the >7 days group) were LTFU and assumed to have high viral load. In summary, a total of 877 (398 participants who started ART on the same-day of HIV diagnosis and 479 participants who started 7 days after their HIV diagnosis) were eligible to be included in our study (Fig 1).

Table 1 describes baseline and follow-up sociodemographic characteristics of participants included in the study in each group. The two groups were similar in marital status, religion and HIV status of partner. The median age of participants was 31 (interquartile range (IQR) 27–39) in the same-day and 33 (IQR 28–41) in the >7 days group.

**Table 1:** Sociodemographic characteristics of study participants by group in Amhara region, Ethiopia, October 20, 2016 to July 18, 2018.

Characteristics	Same-day group	>7 days group	P-value
Sex (n, %)			0.03
Male	172 (43.2)	243 (50.7)	
Female	226 (56.8)	236 (49.3)	
Age in years–Median (IQR)	31 (27.0–39.0)	33 (28.0–41.0)	0.03
Educational status (n, %)			0.02
No Education	98 (24.6)	136 (28.4)	
Primary	109 (27.4)	141 (29.4)	
Secondary	125 (31.4)	103 (21.5)	
Tertiary	45 (11.3)	67 (14.0)	
Missing	21 (5.3)	32 (6.7)	
Marital status (n, %)			0.23
Never married	91 (22.8)	110 (23.0)	
Married	154 (38.7)	198 (41.3)	
Divorced/separated	115 (28.9)	119 (24.8)	
Widow/er	23 (5.8)	21 (4.4)	
Missing	15 (3.8)	31 (6.5)	
Religion (n, %)			0.8
Orthodox	359 (90.2)	436 (91.0)	
Protestant	3 (0.8)	3 (0.6)	
Muslim	31 (7.7)	37 (7.7)	
Missing	5 (1.3)	3 (0.7)	
Place of residence (n, %)			0.003
Within town	341 (85.7)	367 (76.6)	
Out of town	56 (14.1)	109 (22.8)	
Missing	1 (0.2)	3 (0.6)	
Disclosure of HIV+ status* (n, %)			0.002
Disclosed	271 (68.1)	276 (57.6)	
Not disclosed	47 (11.8)	57 (11.9)	
Missing	80 (20.1)	146 (30.5)	
HIV status of partner* (n, %)			0.9
HIV negative	49 (12.3)	65 (13.6)	
HIV positive	92 (23.1)	104 (21.7)	
Unknown	40 (10.1)	47 (9.8)	
No partner	176 (44.2)	206 (43.0)	
Missing	41 (10.3)	57 (11.9)	
Functional status (n, %)			<0.001
Working	387 (97.2)	429 (89.6)	
Ambulatory	11 (2.8)	36 (7.5)	
Bed ridden	0 (0.0)	12 (2.5)	
Missing	0 (0.0)	2 (0.4)	

*In subsequent follow-up visits post-treatment initiation.

HIV, human immunodeficiency virus; IQR, interquartile range.

Baseline and follow-up bio-clinical characteristics of participants in each study group are shown in Table 2. Individuals who were initiated on ART on same-day of and >7 days after HIV diagnosis were similar in BMI and time to 6^th^ and 12^th^ month’s viral load test. While both groups of participants started with a Non-nucleoside Reverse Transcriptase Inhibitors (NNRTI)-based first-line regimen, all (100%, n = 398) of the same-day group were prescribed with a fixed-dose combination (FDC) of Tenofovir (TDF) + Lamivudine (3TC) + Efavirenz (EFV) compared to 96.2% (n = 461) of the >7 days group. Compared to the same-day group, higher proportion of participants in the >7 days group were documented to have ‘good’ ART adherence.

**Table 2:** Bio-clinical characteristics of study participants by group in Amhara region, Ethiopia, October 20, 2016 to July 18, 2018.

Characteristics	Same-day group	>7 days group	P-value
BMI—Median (IQR)	20.2 (18.4–22.4)	19.8 (17.7–22.1)	0.12
CD4 cell count/μl (n, %)			<0.001
<200	49 (12.3)	187 (39.0)	
200–349	30 (7.5)	83 (17.3)	
≥350	77 (19.4)	84 (17.6)	
Missing	242 (60.8)	125 (26.1)	
WHO clinical stage (n, %)			<0.001
Stage I	303 (76.1)	223 (46.6)	
Stage II	63 (15.8)	115 (24.0)	
Stage III	29 (7.3)	119 (24.8)	
Stage IV	3 (0.8)	22 (4.6)	
OI at enrollment (n, %)			<0.001
Yes	27 (6.8)	111 (23.2)	
No	371 (93.2)	368 (76.8)	
CPT at 6-months* (n, %)			<0.001
Yes	107 (26.9)	322 (67.2)	
No	25 (6.3)	9 (1.9)	
Not eligible	266 (66.8)	148 (30.9)	
CPT at 12-months** (n, %)			<0.001
Yes	94 (23.6)	294 (61.4)	
No	32 (8.0)	30 (6.2)	
Not eligible	272 (68.4)	155 (32.4)	
IPT at 6-months* (n, %)			<0.001
Yes	262 (65.8)	197 (41.1)	
No	120 (30.2)	226 (47.2)	
Not eligible	16 (4.0)	56 (11.7)	
IPT at 12-months** (n, %)			<0.001
Yes	258 (64.8)	242 (50.5)	
No	119 (29.9)	185 (38.6)	
Not eligible	21 (5.3)	52 (10.9)	
ARV regimen started (n, %)			<0.001
TDF + 3TC + EFV (FDC)	398 (100.0)	461 (96.2)	
AZT + 3TC + EFV	0 (0.0)	8 (1.7)	
Others	0 (0.0)	10 (2.1)	
Adherence to ART at 6-months* (n, %)			0.01
Good	291 (73.1)	389 (81.2)	
Fair	13 (3.3)	19 (4.0)	
Poor	85 (21.3)	68 (14.2)	
Missing	9 (2.3)	3 (0.6)	
Adherence to ART at 12-months*** (n, %)			0.04
Good	279 (70.1)	376 (78.5)	
Fair	10 (2.5)	10 (2.1)	
Poor	105 (26.4)	89 (18.6)	
Missing	4 (1.0)	4 (0.8)	
Time to first viral load test (months)—Median (IQR)^†^	7 (6.0–9.0)	7 (6.0–10.0)	0.01
Time to 6^th^ month’s viral load test (n, %)			0.47
4- to 5-months	15 (5.9)	25 (8.1)	
6-months	84 (33.2)	92 (29.7)	
7- to 9-months	154 (60.9)	193 (62.2)	
Time to 12^th^ month’s viral load test (n, %)			0.16
10- to 11-months	27 (40.9)	42 (35.9)	
12-months	9 (13.6)	30 (25.6)	
13- to 15-months	30 (45.5)	45 (38.5)	

*Within 6-months of ART initiation.

**Within 12-months of ART initiation.

***Within 6- to 12-months of ART initiation.

ARV, antiretroviral; AZT, Zidovudine; BMI, body mass index; CPT, cotrimoxazole preventive treatment; FDC, fixed dose combination; EFV, Efavirenz; IPT, isoniazid preventive therapy; IQR, interquartile range; 3TC, Lamivudine; OI, opportunistic infection; TDF, Tenofovir; WHO, World Health Organization.

### Viral suppression

By 12-months following ART initiation, 292 (73.4%) of 398 participants in the same-day group and 401 (83.7%) of 479 in the >7 days group had achieved low viral load status (absolute difference = 10.3% (95% CI: 4.9%, 15.8%; *p <* .*001*)). In the adjusted analysis, there was statistically significant difference in low viral load status (adjusted difference = 8.3 (95% CI: 3.5%, 13.0; *p =* .*001*) in the two groups (Table 3).

**Table 3:** Virologic outcomes of participants started ART by group in Amhara region, Ethiopia, October 20, 2016 to July 18, 2018.

Outcomes	No. (%) of participants		Absolute Difference, % (95% CI)	Adjusted Difference (β)^†^, % (95% CI)
	Same-day	>7 days		
**Primary analysis**				
	(n = 398)	(n = 479)		
Low viral load by 12-months^a^	292 (73.4)	401 (83.7)	10.3 (4.9, 15.8)	8.3 (3.5, 13.0)
**Sensitivity analysis**				
	(n = 316)	(n = 344)		
Low viral load by 6-months^b^	232 (73.4)	294 (85.5)	12.0 (5.9, 18.2)	9.1 (2.7, 15.4)
	(n = 319)	(n = 427)		
Low viral load by 12-months^c^ *	292 (91.5)	401 (93.9)	2.4 (1.4, 6.2)	5.1 (1.5, 8.7)
	(n = 398)	(n = 479)		
Low viral load by 12-months ^a^ **	292 (73.4)	401 (83.7)	10.3 (4.9, 15.8)	8.7 (3.8, 13.5)

β, a regression coefficient (Reference group = persons initiated on ART >7 days after HIV diagnosis).

*Excluding LTFU individuals with no viral load result.

**Result from complete-case analysis.

^†^Using IPWRA models—the treatment models adjusted for age, baseline WHO clinical stage, baseline functional status and facility type; and the outcome models adjusted for

^a^age, baseline WHO clinical stage, IPT by 12-month and adherence by 12-month.

^b^baseline WHO clinical stage, CPT by 6-month, IPT by 6-month and adherence by 6-month.

^c^age, baseline WHO clinical stage, CPT by 12-month and adherence by 12-month.

### Sensitivity analysis

The adjusted results of all the sensitivity analyses were similar with the findings of the primary analysis. After adjusting for the covariates, there was statistically significant difference in low viral load status between the two groups in a sensitivity analysis that excluded LTFU participants who did not have viral load test by 12-months (adjusted difference = 5.1% (95% CI: 1.5%, 8.7%; *p =* .*005*)), included viral load tests performed only between 4- and 9-months to estimate low viral load by 6-months (adjusted difference = 9.1% (95% CI: 2.7%, 15.4%; *p =* .*005*)), and applied a complete-case analysis (adjusted difference = 8.7% (95% CI: 3.8%, 13.5%; *p <* .*001*) (Table 3).

## Discussion

This study compared viral suppression (≤1000 copies/mL) of people living with HIV who started ART on the same-day vs >7 days of HIV diagnosis in multiple public health facilities in Ethiopia. We found that individuals who started ART on the same-day of HIV diagnosis had lower viral suppression by 12-months post-ART initiation compared to those who started ART >7 days after their HIV diagnosis.

The suboptimal virologic outcomes we observed in same-day initiated people is contrary to earlier RCTs that demonstrated the efficacy of same-day ART initiation in improving viral suppression at 12-months [11, 15]. This could be due to differences in study settings between RCTs and empirical observational studies. In support of this argument, recent observational studies from routine clinical care in Nigeria [20] and Rwanda [43] showed non-significant difference in viral load suppression among participants who started ART during “test and treat” era compared to those who started before the “test and treat” era. Additionally, a South African study identified similar viral load status among same-day initiators compared to later initiators at 6-months post-ART initiation [19]. In a nutshell, evidence from routine clinical care settings have been emerging to show contrary findings compared to the previous RCTs [11, 15] that had limited generalizability in non-research settings.

The suboptimal viral load status among same-day ART initiators could be explained by various factors. Among these, ART adherence is a key predictor to viral suppression [44]. Studies had showed that suboptimal adherence to ART contributed to a higher risk of virologic failure [45, 46]. This study also demonstrated lower optimal adherence among same-day ART initiators compared to those initiated on ART >7 days later. The accelerated process to immediately initiate on ART the same-day diagnosis likely denies service providers opportunities to adequately [47] address barriers to treatment adherence [48–51]. From patient side, some people may need more time to comprehend the result and cope with the news of being HIV positive before deciding to start a lifelong treatment immediately after diagnosis. Additionally, the suboptimal viral load status among same-day group could be related to a higher attrition in this group that has been published elsewhere [18]. In this study, LTFU individuals with no documented viral load result were considered as having high viral load. Previous studies indicated the need for providing extra-support when adopting same-day initiation [16, 17] to ensure treatment adherence and retention through family- [52], facility- or community-based adherence supports [53] thereby attain viral suppression.

Despite a higher risk of having viral failure among same-day initiators, emerging evidence show an increasing trend in same-day ART enrollment in LIS [16, 17, 24]. Patients with high viral load are at higher risk of developing advanced HIV disease [54] and death [5] and transmitting HIV in the community [7]. This also contribute to the emergency of drug resistance virus for the available limited first-line regimen and it may fuel the increasing trend of pretreatment drug resistance in LIS [55]. On the other hand, limited access to routine viral load monitoring in LIS [16, 20, 23] could affect early detection and management of high viral load. Overall, these could threaten the significant gains achieved in improving quality of life and reducing new HIV infections and AIDS-related deaths in LIS [1]. Therefore, more attention should be given to how to ensure treatment adherence, retention and routine viral load monitoring in the era of universal “test and treat” implementation.

Due to substantial missing data for the 12-months viral load result, we could not separately show the virologic outcomes at exactly 12-month. The reasons for the missing viral load testing could be multifaceted. In Ethiopia, based on our observation and knowledge of the HIV program, challenges related to the availability of limited centralized conventional viral load testing laboratories, frequent viral load machine failure, stockout of reagents, limited days of sample collection and transport, and lack of regular review and timely order of viral load tests by clinicians are some of them to mention. We have used augmented multiple imputation technique [35] to address missing values for the covariates. Our analysis was also limited to the variables available on medical records, and hence some important individual-level factors such as social, psychological, behavioral and mental health issues that could have residual confounding. Finally, in this study individuals who had transferred their care to another health facility within 12-months of ART initiation were not included due to lack of information about their outcomes. Considering a higher chance of discontinuing treatment among transferred-out cases [56] and a little more transferred-out cases among participants who started ART on the same-day [17] or within 2-weeks [20] under universal “test and treat” strategy, the observed low viral load estimates among same-day group may have been overestimated.

This study is one of the few studies conducted to evaluate the universal “test and treat” approach of HIV service delivery in LIS, inimitably focusing on the effect of same-day treatment on virologic outcomes in a multi-center study with a fairly large sample size. We applied strict inclusion criteria to select participants and used double-robust method using IPWRA to compensate for any remaining imbalance of covariates in study groups and generate unbiased estimates [37, 38]. We performed three sensitivity analyses to observe the effect of treatment assuming other scenarios that have resulted in similar findings with the primary analysis.

## Conclusions

Low viral load status among people living with HIV who started ART on the same-day of HIV diagnosis was significantly less during the first year of receiving ART compared to those who started ART >7 days after their HIV diagnosis. Reinforcing ART adherence counseling to those starting ART on the same-day of HIV diagnosis is necessary.

## Supporting information

S1 File Minimal dataset. (XLS)
